# Attitudes toward preimplantation genetic testing and quality of life among individuals with hereditary diffuse gastric cancer syndrome

**DOI:** 10.1186/s13053-022-00239-9

**Published:** 2022-09-02

**Authors:** Ibrahim H. Shah, Erin E. Salo-Mullen, Kimberly A. Amoroso, David Kelsen, Zsofia K. Stadler, Jada G. Hamilton

**Affiliations:** 1 Department of Psychiatry & Behavioral Sciences, Memorial Sloan Kettering Cancer Center, New York, NY USA; 2 Department of Medicine, Memorial Sloan Kettering Cancer Center, New York, NY USA; 3 Department of Medicine, Weill Cornell Medical College, Cornell University, New York, NY USA; 4 Department of Psychiatry, Weill Cornell Medical College, Cornell University, New York, NY USA

**Keywords:** Cancer, Inherited cancer syndrome, Reproduction, Preimplantation genetic testing, Risk

## Abstract

**Background:**

Hereditary Diffuse Gastric Cancer (HDGC) syndrome is an autosomal dominant hereditary cancer predisposition associated with germline pathogenic/likely pathogenic variants in the *CDH1* gene. Identifying early stage HDGC is difficult, and prophylactic measures can be effective in preventing incidence. Preimplantation Genetic Testing (PGT) can provide information about *CDH1* variant status, HDGC risk, and limit familial transmission of *CDH1* variants. To date, however, little is known about the attitudes of individuals with *CDH1* variants towards PGT.

**Methods:**

Given that little is known about the reproductive attitudes of individuals with HDGC, we recruited participants with *CDH1* variants from a familial gastric cancer registry and administered a cross-sectional survey with open- and closed-ended response items. We assessed attitudes regarding PGT and the effect of HDGC on quality of life.

**Results:**

Participants (*n* = 21) were predominantly partnered (61.9%), had a personal cancer history (71.4%), and had biological children (71.4%). Interest in learning about PGT was high; 66.7% of participants were interested in PGT and 90.5% approved of healthcare providers discussing PGT with individuals with *CDH1* variants. Attitudes regarding personal use were varied. Among all participants, 35% would not, 25% were uncertain, and 40% would use PGT. Personal philosophy and preferences for family and reproduction were key factors related to PGT attitudes. HDGC had moderate effects on participants’ quality of life, including social relationships, health behaviors, and emotional experiences including worry about cancer risk and guilt regarding familial implications.

**Conclusion:**

PGT was identified by participants as acceptable for use in a variety of contexts and benefits of reproductive counseling involving PGT may extend beyond *CDH1* carriers to family members’ reproductive behaviors. Dispositions towards PGT are governed by personal philosophy or belief systems. These findings can help guide providers counseling individuals with *CDH1* variants.

**Supplementary Information:**

The online version contains supplementary material available at 10.1186/s13053-022-00239-9.

## Background

Hereditary Diffuse Gastric Cancer (HDGC) syndrome is an autosomal dominant hereditary cancer predisposition syndrome associated with germline pathogenic/likely pathogenic variants in the tumor suppressor E-cadherin (*CDH1*) gene [1, 2]. Although familial clustering of gastric cancer was initially described in a multigenerational Maori family from New Zealand in 1964 [3], Guilford and colleagues first identified *CDH1* germline variants as being a cause of the familial cancer in 1998 [2]. According to a recent investigation of individuals with *CDH1* pathogenic variants, the cumulative incidence of diffuse-type gastric cancer is 70% (95% CI, 59–80%) for males and 56% (95% CI, 44–69%) for females [4]. Due to difficulties in effectively identifying early-stage diffuse-type gastric cancer with currently available surveillance upper endoscopy and random biopsy protocols, many individuals who are found to have a germline *CDH1* pathogenic variant consider prophylactic total gastrectomy [5]. Women who carry germline *CDH1* pathogenic variants also have an increased risk for lobular breast cancer with a risk estimate of 42% (95% CI, 23–68%) [4]. Recommended management for this risk includes increased (earlier age and more frequent) surveillance breast imaging with breast MRI and mammogram, chemoprevention, and some women consider prophylactic bilateral mastectomies [5]. Incomplete penetrance and inter- and intra-familial variability have been noted in HDGC kindreds. More recently, the increasing use of multigene panel testing (MGPT) in clinical practice has also led to uncertainty in the management of patients who present with incidental *CDH1* pathogenic variants. A 2017 study evaluating unexpected *CDH1* variant identification during MGPT illustrated that there may be a substantial number of persons with *CDH1* pathogenic variants who do not meet diagnostic testing criteria and would otherwise not have been identified [6]. Given that current penetrance estimates have been derived largely from families ascertained by stringent HDGC guidelines, it is unknown whether such incidentally identified results may represent less penetrant mutations of *CDH1*. Updated clinical guidelines for the management of HDGC acknowledge that while prophylactic total gastrectomy remains the gold standard, advancements in access and utility of MGPT and an evolving understanding of *CDH1* penetrance have led to increasing confidence that other prophylactic measures like endoscopic surveillance might be considered for patients in this context [5]. These findings underscore the difficulties both patients and providers may face in clinical management and prophylactic/surveillance decision-making with respect to germline *CDH1* findings [7].

Given the heritable nature of HDGC, evolving data on germline *CDH1* variants and their associated risks, and difficulties with respect to clinical management and decision-making for patients in this setting, a role exists for reproductive technologies that could control the transmission of genetic risk to future generations. Preimplantation genetic testing for monogenic conditions (PGT-M, referred to hereafter as PGT) is one such reproductive technology. PGT involves sampling DNA from an in vitro fertilization (IVF)-created embryo followed by genetic testing for particular genetic alterations. IVF-PGT was initially used for childhood-onset conditions such as cystic fibrosis [8]. However, it is now increasingly used for adult-onset conditions, including hereditary cancer predisposition syndromes such as hereditary breast and ovarian cancer syndrome (i.e., germline *BRCA1*/*BRCA2* variants) and Lynch syndrome (previously known as Hereditary Non-Polyposis Colorectal Cancer/HNPCC due to germline *MLH1*, *MSH2*, *MSH6*, *PMS2*, or *EPCAM* variants) [9]. Prior studies have investigated attitudes of cancer predisposition variant carriers towards the use of IVF-PGT [10–21]. In these investigations, a generally favorable attitude towards PGT, consisting of a desire to learn about and willingness to consider PGT, was identified among respondents. To date, however, little is known about the attitudes of individuals with *CDH1* variants towards PGT. In a 2017 report of qualitative interviews with 35 high risk individuals (defined as having a confirmed *CDH1* variant or having undergone prophylactic total gastrectomy) from the United Kingdom-based Familial Gastric Cancer Study, again a generally favorable view of reproductive genetic testing was identified [21]. A preference for PGT over prenatal diagnosis (PND) through chorionic villus sampling or amniocentesis was noted due to the ability to potentially avoid difficult decision-making regarding elective pregnancy termination that can be associated with PND. Additionally, some interviewees reported anxiety and skepticism about IVF. Participants were also concerned about the need for planned reproduction and anticipated difficulty communicating this to partners. Interviewees noted differences in the information provided by healthcare providers, which may have influenced observed differences in opinion regarding PGT. This suggests that further data are needed to elucidate the PGT informational needs among this patient population, and that this information should inform genetic counseling practices for HDGC [21].

To further contribute to the literature on reproductive genetic technology and HDGC, we collected quantitative and qualitative survey data from individuals with confirmed *CDH1* variants. Here, we describe findings regarding these individuals’ opinions on reproductive options and their experiences with and knowledge of PGT. We also provide data regarding their opinions of how a diagnosis of HDGC has impacted their reproductive choices and broader quality of life.

## Methods

### Participants

Study participants were recruited from the Early Onset and Familial Gastric Cancer Registry, a multi-institutional prospective registry created to identify individuals with gastric cancer at high risk for a familial predisposition syndrome to gastric cancer. Registry eligibility was limited to patients with 1) a known diagnosis of early-onset (i.e., before age 50 years) gastric cancer; or 2) gastric cancer and a family history of gastric cancer in at least one first-degree relative or two second-degree relatives; or 3) a known *CDH1* variant. The subgroup of registry participants who were eligible for the present survey study include those who were enrolled to the registry by Memorial Sloan Kettering Cancer Center (MSK) between 2005 and January 2013, were age 18 years or older, had a pathogenic/likely pathogenic *CDH1* variant, and had agreed to be contacted for future studies.

### Procedures

Survey participants were recruited by study staff via phone. Interested participants provided verbal informed consent and were mailed the study survey with a pre-paid envelope for return to study staff. This study was approved by the MSK Institutional Review Board.

### Measures

The survey consisted of self-reported items developed with guidance from institutional experts in clinical genetics, gastrointestinal oncology, fertility, and behavioral sciences. Medical terminology, such as PGT (referred to as preimplantation genetic diagnosis and PGD in study materials), pathogenic/likely pathogenic *CDH1* variants (referred to as mutations in study materials), IVF, donor gametes, and other terms were defined for the participants in readily comprehensible language. The 50 (43 closed-ended and 7 open-ended) item survey (see Additional File 1) took approximately 25 min to complete and collected information on the following topics:

#### Sociodemographics

Age, gender, race/ethnicity, education, marital status, and income were assessed.

#### Clinical history

Personal cancer history (yes/no response) and personal cancer diagnoses (open-ended) were assessed. Uptake of total gastrectomy was also assessed (response options: “Yes, I have undergone a prophylactic total gastrectomy,” “Yes, I have undergone total gastrectomy that was initially thought to be prophylactic, but gastric cancer cells were found upon surgical pathology review,” “Yes, I have undergone a total gastrectomy because of a previously identified gastric cancer,” “ No, I have not undergone total gastrectomy”). Family cancer history in terms of number of first-degree relatives diagnosed with a HDGC-associated cancer and deceased due to a HDGC-associated cancer (response scale from “0” to “11 + ”) were also assessed.

#### Reproductive history

Participants’ biological parental status (yes/no), and age of any children (open-ended) were assessed. Knowledge of children’s *CDH1* variant status (positive or negative for the familial *CDH1* variant) was assessed (response options: “Yes, I know all of my biological children's mutation statuses,” “I know some of my biological children's mutation statuses,” “No, I do not know any of my biological children's mutation statuses”, “Other”). Items also assessed whether participants or their partners were diagnosed with infertility; used donor gametes, and if so, used donor gametes to eliminate the risk of a *CDH1* variant in their children; used IVF; used PGT; had adopted children, and if so, if they adopted to eliminate the risk of a *CDH1* variant in their children (yes/no responses).

#### Attitudes toward PGT

Items assessed whether participants want to have (more) biological children now or in the future, had heard about PGT prior to participating in the survey, believe it is an acceptable practice for healthcare providers to inform individuals who have *CDH1* variants about the availability of PGT, had previously considered using PGT, believe PGT is acceptable for conditions that occur in childhood, and believe that PGT is acceptable for families with *CDH1* variants (yes/no responses). Participants indicated their level of interest in learning more about PGT (Likert-type scale ranging from 0 = “not interested” to 4 = “very interested”). All participants were also asked about their likelihood of using PGT for the *CDH1* variant (in the future if they wanted to have more biological children, or if this technology had been available if they have already completed their family) on a Likert-type scale ranging from 1 = ”definitely not” to 5 = ”definitely.” Participants were asked if after completing the survey they would discuss the availability of PGT with a family member who has a risk to have a child with the *CDH1* variant (Likert-type scale ranging from 1 = ”definitely not” to 5 = “definitely” with 6 = “not applicable-nobody appropriate in my family”). Finally, two open-ended items assessed their main concerns or worries about PGT and what they perceive as the main benefit of PGT.

#### Impact of HDGC on quality of life

Participants indicated how often they worry about their chances of developing gastric and/or breast cancer (Likert-type scale ranging from 1 = “not at all/rarely” to 4 = “almost all the time”). Participants rated how severely HDGC syndrome affected their overall health and well-being (scale of 1 = “not severe” to 10 = “most severe”). Participants indicated whether they experience guilt related to HDGC syndrome affecting their family members (yes/no). Items also assessed how satisfied participants are with their quality of life and whether having HDGC syndrome caused them to alter important life decisions (Likert-type scale ranging from 1 = “uncertain” to 6 = “very much”). Similarly, an item assessed whether having HDGC syndrome affected their current or future reproductive decisions (Likert-type scale ranging from 1 = “uncertain” to 6 = “very much” with 7 = ” not applicable”). Finally, two open-ended items assessed how HDGC syndrome has caused participants to alter important life decisions and has affected their current or future reproductive decisions.

### Statistical analysis

Descriptive statistics were computed to summarize participant responses to all closed-ended items. To analyze the qualitative data from open-ended items about attitudes toward PGT and the impact of HDGC on quality of life, we used qualitative inductive analysis [22–26]. This process of narrative review, interpretation, and consensus discussions was performed on participants’ verbatim responses to the open-ended items. The Framework analytic induction approach [22, 27, 28] was used, which is appropriate for analyses that begin deductively and aim to develop a thematic codebook and identification of key themes. Participant responses were independently coded and categorized for thematic content by two reviewers (J.G.H. and K.A.), and the reviewers subsequently met in consensus meetings to jointly compare and reconcile differences to maximize internal validity.

## Results

### Participant characteristics

Thirty-seven living patients with confirmed *CDH1* variants were eligible for the survey; of these, 22 patients consented to the present study and 21 returned a completed survey (57% response rate). Characteristics of the study sample (*n* = 21) are summarized in Table 1. The majority was female (81%), white/Caucasian (90.5%), and were married or partnered (61.9%).

**Table 1:** Participant characteristics (*n* = 21)

	*n* (%)
Sociodemographics	
Age, years (*M* ± *SD)*	51.4 ± 13.3; range: 30–75
Gender (Female)	17 (81.0)
Race (White/Caucasian)	19 (90.5)
Marital status	
Married or partnered	13 (61.9)
Divorced or separated	5 (23.8)
Single	3 (14.3)
Educational attainment	
Some college	4 (19.0)
College graduate	6 (28.6)
Post-graduate	11 (52.4)
Annual household income^a^	
< $50,000	1 (4.8)
$50,000-$100,000	9 (42.9)
$100,000-$200,000	6 (28.6)
> $200,000	4 (19.0)
Clinical history	
Personal cancer history (Yes)	15 (71.4)
Personal cancer diagnosis^b^	
Lobular breast cancer	6 (28.6)
Stomach cancer	13 (61.9)
Other cancers	3 (4.8)
Number of 1^st^ degree relatives with a HDGC cancer (*M* ± *SD)*	3 ± 2.8; range: 0–8
Number of 1^st^ degree relatives who died from a HDGC cancer (*M* ± *SD)*	1 ± 2.4; range: 0–8
Total gastrectomy^a^	
Yes, prophylactic	8 (38.1)
Yes, prophylactic but occult gastric cancer detected	5 (23.8)
Yes, due to gastric cancer	3 (14.3)
No	4 (19.0)
Reproductive history	
Have biological children (Yes)	15 (71.4)
Know biological children's *CDH1* mutation status^c^	
Yes, know all children’s statuses	6 (40.0)
Yes, know some children’s statuses	5 (33.3)
No	3 (20.0)
Have adopted children to eliminate the risk of a *CDH1* mutation	0 (0)
Have used donor gametes (Yes)	0 (0)
Have used PGT and in vitro fertilization (Yes)	1 (4.8)
Want (more) biological children (Yes)^a^	2 (9.5)

^a^ Data were missing for one participant; percentages calculated out of total sample

^b^ Participants could endorse more than one cancer type

^c^ Percentages computed out of a total of 15 participants with biological children; 1 participant declined to respond

Most participants (71.4%) had a personal cancer history. Most had biological children (71.4%; child ages ranged from 1–52 years). A minority of participants wanted to have (more) biological children now or in the future (9.5%). None of the participants or their partners had previously used PGT.

### Attitudes toward PGT

Among all participants, 66.7% had previously heard of PGT, although only 14.3% of the sample had previously considered using PGT. Most participants (66.6%) were “somewhat interested” or “very interested” in learning more about PGT. Participants held generally favorable attitudes about the availability of PGT. The majority (90.5%) believed that it was acceptable for healthcare providers to inform individuals who have a *CDH1* variant about the availability of PGT. Furthermore, 76.2% of participants believed that PGT is acceptable for both conditions that occur during childhood and for families with *CDH1* variants (see Table 2).

**Table 2:** Participant attitudes about PGT, HDGC, and quality of life (*n* = 21)

	*n* (%)
Had you heard about PGD prior to participating in this survey?	
Yes	14 (66.7)
No	7 (33.3)
Have you previously considered using PGD?	
Yes	3 (14.3)
No	16 (76.2)
Missing	2 (9.5)
Level of interest in learning more about PGD	
Not interested	7 (33.3)
Uncertain	0 (0)
Somewhat interested	7 (33.3)
Very interested	7 (33.3)
Do you believe it is an acceptable practice for healthcare providers to inform individuals who have *CDH1* gene mutations about the availability of PGD?	
Yes	19 (90.5)
No	2 (9.5)
In general, do you believe that PGD is acceptable for conditions that occur during childhood?	
Yes	16 (76.2)
No	4 (19.0)
Missing	1 (4.8)
Do you believe that PGD is acceptable for families with *CDH1* gene mutations?	
Yes	16 (76.2)
No	4 (19.0)
Missing	1 (4.8)
How often do you worry about your chances of developing gastric and/or breast cancer (again)?	
Not at all/ Rarely	7 (33.3)
Sometimes	5 (23.8)
Often	7 (33.3)
Almost all the time	2 (9.5)
Do you experience guilt related to HDGC syndrome affecting your family members?	
Yes	12 (57.1)
No	9 (42.9)
On a scale of 1 (not severe) to 10 (most severe), how severely has HDGC syndrome affected your overall health and well-being? (*M* ± *SD)*	6.0 ± 2.7; range: 2–10

Note that because the original survey used the terminology “preimplantation genetic diagnosis; PGD,” this language has been retained in the item wording

**Fig. 1:**
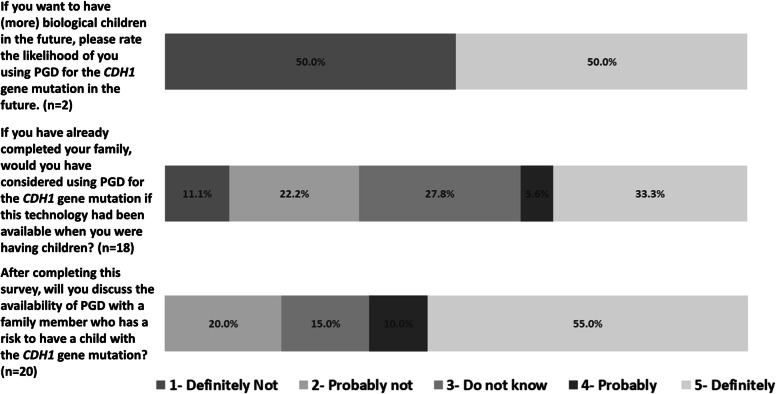
Participant attitudes regarding PGT. Note that because the original survey used the terminology “preimplantation genetic diagnosis; PGD,” this language has been retained in the item wording

Participants expressed a wide range of opinions regarding their own likelihood of using PGT. Among the two participants who wanted to have biological children in the future, interest in using PGT for *CDH1* variants in the future was evenly split with one (50%) being “definitely not” likely to use the technology and one (50%) being “definitely” likely to use the technology. Among the remaining 18 respondents (note one missing response) who had already completed their families, their likelihood of having used PGT if the technology had been available when they were making their own reproductive decisions also varied. Slightly more than a third (38.9%) of these participants would have been likely to use this technology, 27.8% were undecided, and 33.3% would not have used this technology (see Fig. 1). Finally, the majority (65.5%) of all participants would “definitely” or “probably” discuss the option of PGT with a family member at risk of having a child with a *CDH1* variant.

Qualitative analysis of the open-ended survey items revealed multiple factors that informed and shaped participants’ attitudes about the use of PGT (Table 3). Most commonly, participants’ responses included a discussion of a *personal philosophy* or belief system that guided and justified their reproductive decisions and attitudes about the appropriateness of PGT. Such beliefs included their attitudes about uncertainty and the perceived appropriateness or importance of either embracing or controlling possible reproductive outcomes. Responses also frequently described the influence of *God, religion, or morality* on these reproductive choices, including attitudes about what a higher power may choose for them, as well as innate feelings of what is ethically right or wrong. An additional factor relevant to participants’ perspectives about PGT was their *personal preferences for family and reproduction* such as preserving the ability to have a biological child.

**Table 3:** Codes, definitions, number of times code was applied, and illustrative participant responses regarding attitudes about the use of PGT

Code	Definition	Number^a^	Illustrative Participant Response
Personal philosophy	Individuals describe a personal philosophy or belief system (e.g., acceptance, embracing uncertainty, personal boundaries) that justifies their reproductive decisions	13	“Life is what it is. You take the good with the bad!” [ID 14]
God, religion, and morality	Individuals discuss a trust in God and/or how the use of PGT may violate their religious or moral beliefs	10	“I believe in trusting God. If my children have it then I will know how to handle it.” [ID 20]
Eliminate mutation	Individuals describe the benefit of preventing or eliminating a pathogenic mutation in future generations from use of PGT	9	“Stopping the transmission of the mutation to next generation.” [ID 18]
Minimizing suffering or anxiety	Individuals describe a benefit of minimizing personal or children’s suffering and anxiety from use of PGT	5	“I think if you can help your children to avoid the pain of cancer, then one should do it. Especially for your children.” [ID 9]
Personal preferences for family and reproduction	Individuals describe their personal values and preferences regarding reproduction and having biological children	5	“I would have either adopted or not had children.” [ID 12]
Concerns about safety and technology	Individuals express concerns about the safety or accuracy of genetic/reproductive technology	4	“Sometimes results may be a false positive, my brother took the gene test and every time the result was different 3–4 times.” [ID 1]
Cost	Individuals describe costs and financial implications of PGT	2	“Cost, safety for embryos.” [ID 17]
Age	Individuals describe their age as a factor in their decisions about PGT and reproduction	2	“Because we are too old.” [ID 4]
Improving health	Individuals express a benefit of improved health for children from PGT	2	“Longer, disease-free (hopefully) life.” [ID 15]
Knowledge	Individuals express a benefit of improved knowledge from PGT	1	“Knowledge.” [ID 14]

*PGT* Preimplantation genetic testing

^a^ Out of 38 total responses

Several perceived benefits of PGT were described by participants, with the most common responses reflecting the ability to *eliminate mutations* known to be pathogenic (e.g., *CDH1*) and to *minimize suffering or anxiety* experienced by themselves and their children. Less frequently noted benefits included the ability to *improve health* of future children and *knowledge* that could be gained through PGT. A few barriers or concerns regarding PGT were also noted. Most commonly, responses described *concerns about safety and technology* that may limit the effectiveness or reliability of PGT in ensuring an anticipated, promised outcome. A few participants also expressed concerns about the *cost* of the technology. Finally, two participants perceived that their *age* would preclude them from employing PGT (it is worth noting that both of these participants expressed a disinterest in having additional biological children).

### Impact of HDGC on quality of life

Survey items also assessed participants’ perceptions of the broad impact of HDGC on their lives. Although a third (33.3%) of participants noted that they were “not at all/rarely” worried about their chances of developing gastric and/or breast cancer, 57.1% reported experiencing such worries “sometimes” or “often”, and 9.5% worried about this possibility “almost all the time.” Furthermore, participants believed that HDGC had exerted a moderately severe effect on their overall health and well-being (*M* = 6.0, *SD* = 2.7 on a 1–10 scale). In addition, a slight majority (57.1%) of participants reported experiencing guilt related to HDGC syndrome affecting their families (see Table 2).

**Fig. 2:**
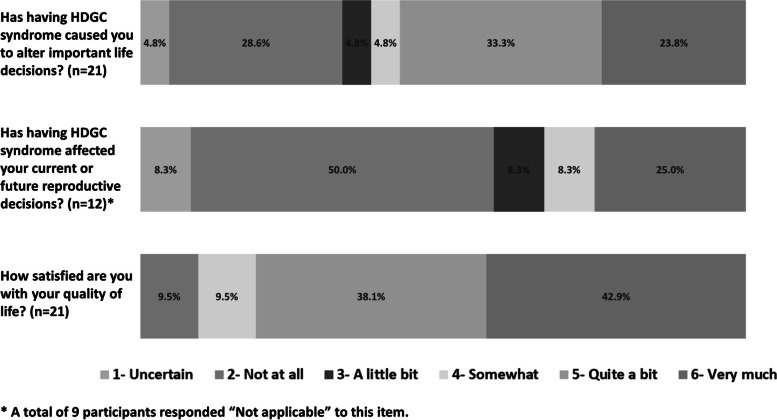
Participant perceived impact of HDGC on quality of life

As depicted in Fig. 2, participants expressed diverse opinions about the extent to which HDGC has influenced their lives. Most (66.7%) believed that HDGC had at least some effect (responses ranging from “a little bit” to “very much”) on their important life decisions. However, only 41.6% of participants believed that HDGC had an effect (responses ranging from “a little bit” to “very much”) on their current or future reproductive decisions. Overall, participants were generally satisfied with the quality of their lives, with 81% reporting that they were “quite a bit” or “very much” satisfied.

Qualitative analysis revealed a range of ways in which HDGC had an impact on the lives of study participants (Table 4). Many described the effects of HDGC on their *reproduction* choices and outcomes, predominantly in terms of choosing against having biological children or seeking information or reproductive technologies to minimize risks to offspring. Many participants also described effects on their *social* relationships and functioning, including adverse impacts on work decisions and roles, strains on romantic relationships, and influencing topics of conversation with family and friends. Participants also described changes in their *cancer screening, prevention, and health behaviors* that were attributable to their diagnosis of HDGC syndrome. Several participants discussed the ways in which HDGC affected their *overall health*, including their physical and cognitive functioning. Participants also expressed *concerns about children,* namely involving their physical and emotional well-being, which arose because of HDGC syndrome. Finally, several participants described the implications of HDGC for their *emotional distress*, noting that they had experienced increased anxiety, worry, and depression.

**Table 4:** Codes, definitions, number of times code was applied, and illustrative participant responses regarding life impact of HDGC syndrome

Code	Definition	Number^a^	Illustrative Participant Response
Reproduction	HDGC syndrome has caused individuals’ to make decisions, seek information, or pursue medical interventions relevant to reproduction	12	“I am a gay male, so option = bio or adopt. Being diagnosed with HDGC just made it certain I would not try to have my own biological kids.” [ID 18]
Social	HDGC syndrome has caused changes in individuals’ interpersonal relationships (e.g., marriage, work) and social functioning	10	“Broke up relationship I was in at the time because I decided kids were not going to be an option.” [ID 13]
Cancer screening, prevention, and health behaviors	HDGC syndrome has caused individuals to pursue and/or be more mindful about cancer screenings, prevention interventions (e.g., gastrectomy), and overall health	7	“Significant impact on life – having gastrectomy, decision to pursue PGD in future for childbearing, and weighs into decisions about job change (can't take different job as the health benefits aren't as good as my current job). Decisions regarding breast screening; prophylactic mastectomy in the future?” [ID 17]
Overall health	HDGC syndrome has had an impact on individuals’ health (e.g., diet, cognitive functioning, energy, pain)	6	“Can't eat regular meals, energy levels are not what they used to be.” [ID 1]
Concerns about children	HDGC syndrome has caused individuals to worry about their children's and family’s future health and wellbeing	5	“Honestly, I think I have made my way through life... assuming that I would have a shorter lifespan than normal. Retirement was not something I planned for. As I age, I think I need to think about that more. Hopefully, I will be around to help my daughter deal with a life like mine.” [ID 11]
Emotional distress	Living with HDGC syndrome has created feelings of distress such as depression or anxiety	4	“Having cancer resulted in my divorce. I worry about my children all the time, which has caused stress and severe depression.” [ID 3]

*HDGC* Hereditary diffuse gastric cancer

Note that because the original survey used the terminology “preimplantation genetic diagnosis; PGD,” this language has been retained in participant quotes

^a^ Out of 24 total responses

## Discussion

This study evaluated attitudes of individuals with *CDH1* variants towards PGT and their perceptions of how HDGC affects their overall quality of life. The integration of quantitative and qualitative data revealed several key findings. Although most participants had previously heard of PGT, few had considered using this technology. Most participants already had biological children, and very few wanted any or more biological children in the future. Nonetheless, PGT was frequently identified by participants as acceptable for use in a variety of contexts. For instance, almost all participants found it acceptable for healthcare providers to discuss PGT with individuals with *CDH1* variants and a majority also believed PGT was acceptable for conditions occurring in childhood or for families with *CDH1* variants. Most also displayed a high degree of interest in additional information about PGT. These findings are consistent with and further support previous literature that demonstrates overall acceptability and utility of PGT amongst the general public [29], and suggest that guidelines that include a recommendation for reproductive counseling as part of the management of patients with *CDH1* variants could be well received [30].

Although participants often expressed a willingness to discuss PGT with at-risk relatives, their views were less consistent regarding their personal use of PGT. Amongst the few participants considering having a biological child in the future, participants were divided between being either very likely or very unlikely to personally use PGT. Attitudes about hypothetical use among those participants who had completed their families were more varied, although a slight majority expressed a likelihood to have used PGT if it had been available to them. Previous research on the reproductive concerns of females with *BRCA1/BRCA2* variants (of whom 61% had biological children and 47% were unlikely to have more children) demonstrated that individuals may possess a high degree of concern regarding mutational status or exhibit interest in PGT, but ultimately have strong mixed views on their own adoption of PGT [31]. The investigators noted that due to these concerns, only 13% of respondents were interested in using PGT to select embryos without the familial *BRCA1/BRCA2* variant. Additionally, respondents without children were more likely to consider using PGT and other available assisted reproductive methods than those with children. Consistent with this previous research, the present findings suggest that although reproductive counseling may have the most impact for people without children and/or those considering children, there may also be benefits to discussing this topic with all *CDH1* carriers given their generally high level of willingness to discuss PGT with family members, which may in turn affect familial reproductive decision-making.

Inductive qualitative analysis further revealed that participants with *CDH1* variants had a personal philosophy or belief system that guided their disposition towards PGT. This belief system frequently referenced the influence of spirituality or morality, and participants often had concrete personal values and preferences about reproduction that informed their perspectives on the benefits and drawbacks of PGT. These findings are consistent with a narrative review of the literature about patient attitudes and decision-making factors regarding PGT, which found that a complex set of themes including concrete, personal, and ethical factors influence decision-making [32]. Major themes identified amongst patients included moral judgments such as personal attitudes on the moral status of an embryo and the moral acceptability of PGT. Some patients reported a sense of responsibility to utilize PGT and others reported feelings of conflict with their personal ethics or a sense of “playing God.” Additionally, patients frequently considered the use of PGT through the lens of value-based judgments and preferences both as individuals and as couples. This review also highlighted a limitation of past work in terms of the lack of a mixed-methods data collection approach. Our research, integrating quantitative and qualitative survey data, begins to address this gap in the literature and corroborates the conclusions drawn from this review. Given the strong impact that these factors have on patient preferences for PGT, these findings highlight the need for recognizing and integrating patient attitudes and value-based preferences into the counseling and care that genetic counselors and other healthcare providers deliver to individuals with *CDH1* variants.

Participants viewed the main benefits of PGT to be the potential elimination of variants like *CDH1* and the minimization of suffering and anxiety. Primary disadvantages and barriers to PGT included concerns about safety and technology, and two participants expressed concerns regarding costs of the technology. This result contrasts with research indicating that cost is of considerable concern amongst those who engage in PGT [32, 33], and may be due to relatively few participants in our sample actively considering having biological children in the future and thus having less knowledge or consideration of the financial and logistical implications of PGT. Research does indicate that these concerns are evolving [34] and may constitute less of a barrier to PGT implementation as knowledge regarding PGT increases, costs and accessibility improve, and the perception of PGT shifts from an unknown and futuristic procedure to a known diagnostic tool in reproductive decision-making. Together, our findings and those of previous qualitative studies [34] demonstrate the utility of a concerted effort amongst clinicians, genetic counselors, and other healthcare providers to be prepared to discuss both the personal and practical aspects of PGT with patients with appreciation for the complexity of decision-making for those considering assistive reproductive measures. Additionally, established guidelines from professional or institutional entities that acknowledge these nuances are likely to be effective aides for providers working with this patient population [35].

Important conclusions regarding the broader impact of HDGC on patients’ quality of life can also be drawn from this study. Although a large majority reported satisfaction with their lives, most participants did report some degree of worry regarding their likelihood of developing gastric or breast cancer. Participants frequently asserted that HDGC had some effect on their lives and decisions, with qualitative data most often describing implications for a variety of social situations, cancer screening and prevention, and general health behaviors. Half of the sample also noted an impact of HDGC on reproductive decision-making, including direct and indirect implications for relationship functioning, preferences against having biological children, and motivations to prevent suffering in future generations. Past research has demonstrated that knowledge of a potential or actual HDGC diagnosis and/or *CDH1* variant can affect decision-making with regards to health and social behaviors including reproductive choices [36]. Additionally, many participants expressed guilt related to HDGC syndrome affecting their families. Qualitative data further highlighted the presence of emotional distress and concern for children and families. These findings are similar to those of Hallowell et al. [36], whose research sought to examine decision-making regarding prophylactic total gastrectomy amongst individuals at high-risk for HDGC. These individuals reported feelings of guilt related to HDGC syndrome and their families, as well as an increased family burden.

### Strengths and limitations

The cross-sectional design of this study allowed for a “snapshot” perspective into the minds of patients with *CDH1* variants, and the high response rate indicated participants were willing to openly share their feelings regarding PGT and HDGC. Limitations of this study include reliance on investigator-designed measures, a relatively small sample size that prevented more complex statistical analyses, and the age of the data collected and used for analysis. Data were collected from registry participants in 2014, and as such, their responses may not reflect recent advances in awareness and access to resources like PGT or enhanced *CDH1* surveillance methods. Participants were also predominantly white, well educated, married, cancer-affected, female, and their views may not accurately reflect the larger population of interest. Additionally, participants were recruited from an Early Onset and Familial Gastric Cancer Registry, and as such, these results may not be translatable to those who have a genetic diagnosis but no personal or familial history of HDGC. Patients in this setting (e.g., those with incidentally identified *CDH1* variants from use of MGPT) may have a different risk profile, and associated management and prophylactic considerations compared to our study sample. These differences may alter attitudes and perceptions towards the possibility of passing a *CDH1* pathogenic variant to their children and the use of PGT as a prophylactic measure.

Lastly, it is important to acknowledge that our sample (*M* = 51.4 years old) may not reflect the typical age of patients considering future reproduction. Our sample included only two individuals actively considering future reproduction, and while many participants (*n* = 15) already had biological children, most respondents offered their hypothetical and/or retrospective perspectives on PGT and reproductive decision-making.

### Future research

Future research would benefit from a validated, structured instrument for evaluating PGT decision-making [32], and should recruit a large, diverse sample of patients at risk of HDGC who are considering having biological children. Future research could also explore the variation that appears to exist within patients’ attitudes for the use of PGT and reproductive technology for members of society, their families, and themselves, and further examine how reproductive decisions are contextualized within the broader set of decisions regarding health behaviors faced by individuals with *CDH1* variants. Future studies could further evaluate attitudinal disposition towards PGT through the lens of patients’ belief systems, including their religious, moral, and family frameworks. Given research suggesting that individuals’ opinions about the personal use of PGT may be a function of age [34], such efforts should also examine how age and the varying social perceptions that come with age affect individuals’ varying perspectives and practices regarding the personal use of PGT, the use of PGT by family members, as well as contrasting perspectives on HDGC and quality of life evident in this study and previous research. Together, such efforts would provide a more nuanced understanding of the barriers to PGT and complexities of reproductive decision-making, as well as the larger implications of HDGC on quality of life for individuals and families affected by this syndrome.

## Conclusion

Among patients with *CDH1* variants, PGT was identified as acceptable for use in a variety of contexts. Familial concerns shape not only patients’ reproductive decisions, but also have implications for their quality of life as they navigate their elevated cancer risks. Reproductive counseling involving PGT may have benefits that extend beyond *CDH1* carriers to help inform or shape their family members’ reproductive behaviors. These findings can help guide providers counseling individuals with *CDH1* variants.

## Supplementary Information

**Additional file 1. **Survey Items.

## Data Availability

The data that support the findings of this study are available from the corresponding author upon reasonable request.

## Abbreviations

HDGC: Hereditary Diffuse Gastric Cancer syndrome
IVF: In vitro Fertilization
PGT: Preimplantation genetic testing
PND: Prenatal diagnosis
MGPT: Multigene panel testing
