# Supplementary material for: Attitudes toward preimplantation genetic testing and quality of life among individuals with hereditary diffuse gastric cancer syndrome
Source: Hered Cancer Clin Pract. 2022 Sep 2;20:31. doi: 10.1186/s13053-022-00239-9 (PMC9440538; doi:10.1186/s13053-022-00239-9)
Supplement: Supplementary file 1 — Additional file 1. Survey Items. [file 13053_2022_239_MOESM1_ESM.pdf]

Hereditary Cancer in Clinical Practice

Additional File 1:

Survey Items

Attitudes Toward Preimplantation Genetic Testing and Quality of Life among Individuals with Hereditary Diffuse Gastric Cancer Syndrome

Ibrahim H. Shah, BA

Erin E. Salo-Mullen, MS, MPH, CGC

Kimberly A. Amoroso, MS, CGC

David Kelsen, MD

Zsofia K. Stadler, MD

Jada G. Hamilton, PhD, MPH

Corresponding author: Jada G. Hamilton, Memorial Sloan Kettering Cancer Center, 641 Lexington Avenue, 7th floor, New York, NY 10022. Phone: 646-888-0049, Fax: 212-888-2584, Email: hamiltoj@mskcc.org.

DEMOGRAPHIC INFORMATION:

What is your gender?

- Male
- Female

What is your current age? ____________

What do you consider to be your race/ethnicity?

- Caucasian
- Asian
- Black or African
- Hispanic
- Other:____________

What is your current relationship status?

- Single
- Married / Life Partner
- Separated / Divorced
- Widow / Widower
- Other:____________

Please describe your highest level of education:

- Did not complete high school
- High school graduate
- Attended some college, but did not graduate
- College graduate
- Post-graduate

Please describe your current household income:

- Less than $50,000 per year
- Between $50,000-100,000 per year
- Between $100,000-200,000 per year
- More than $200,000 per year

Which, if any, of the following religions do you identify with as being a member?

- Buddhist
- Catholic
- Eastern Orthodox
- Hindu
- Jewish – Ashkenazi
- Jewish – Sephardic
- Jewish (other)
- Latter Day Saints or Mormon
- Muslim
- Protestant
- Seventh Day Adventist
- None
- Other:_______________________

Please rate the impact that religion has on your overall decision-making:

- Not at all
- Somewhat
- Moderately strong
- Very strong

PERSONAL & FAMILY HISTORY INFORMATION:

Personal Cancer History:

Have you been diagnosed with cancer?:

- o Yes
- o No

If yes, what type of cancer were you diagnosed with and how old were you at diagnosis?

Type #1:_________________________ Age at #1 diagnosis: _________________________

Type #2:_________________________ Age at #2 diagnosis: _________________________

Type #3:_________________________ Age at #3 diagnosis: _________________________

In what year did you have *CDH1* genetic testing? _________________________

Have you undergone total gastrectomy?

- o Yes, I have undergone a prophylactic total gastrectomy.
- o Yes, I have undergone total gastrectomy that was initially thought to be prophylactic, but gastric cancer cells were found upon surgical pathology review.
- o Yes, I have undergone a total gastrectomy because of a previously identified gastric cancer.
- o No, I have not undergone total gastrectomy.

If yes, how old were you when you underwent total gastrectomy? _________________________

Family Cancer History:

How many (if any) of your first-degree relatives (parents, siblings, children) have been diagnosed with a HDGS-associated cancer (i.e. diffuse gastric cancer or lobular breast cancer)?

0 1 2 3 4 5 6 7 8 9 10 11+

If you have had first-degree relatives who have been diagnosed with HDGC-associated cancers, hold old were these family member when they were diagnosed with their (first) cancer?

Family Member #1:__________ Family Member #2: __________

Family Member #3:__________ Family Member #4: __________

Family Member #5:__________ Family Member #6: __________

Family Member #7:__________ Family Member #8: __________

Family Member #9:__________ Family Member #10: __________

Family Member #11:__________ Family Member #12: __________

How many (if any) of your first-degree relatives (parents, siblings, children) have died from a HDGC-associated cancer (i.e. diffuse gastric cancer or lobular breast cancer)?

0 1 2 3 4 5 6 7 8 9 10 11+

If you have had first-degree relatives who have died from a HDGC-associated cancer, how old were these family members when they have died from their cancers(s)?

Family Member #1:__________ Family Member #2: __________

Family Member #3:__________ Family Member #4: __________

Family Member #5:__________ Family Member #6: __________

Family Member #7:__________ Family Member #8: __________

Family Member #9:__________ Family Member #10: __________

Family Member #11:__________ Family Member #12: __________

Personal Reproductive History:

Do you have biological children (conceived using your and your partner's egg and sperm)?

- o Yes
- o No

If yes, what are the ages of the children?

Child #1: __________ Child #2: __________ Child #3: __________

Child #4: __________ Child #5: __________ Child #6: __________

Do you know your biological children's *CDH1* mutation status (i.e. positive or negative for the familial *CDH1* mutation)?

- o Yes, I know all of my biological children's mutation statuses.
- o I know some of my children's mutation statuses.
- o No, I do not know any of my biological children's mutation statuses.
- o Other:____

Have you or your partner experienced 2 or more pregnancy losses (i.e. miscarriages)?

- o Yes
- o No

Have you or your partner been diagnosed with infertility?

- o Yes
- o No

Have you or your partner used donor gametes?

- o Yes
- o No

If yes, did you use donor gametes to eliminate the risk of a *CDH1* mutation in your children?

- o Yes
- o No

Have you or your partner used in-vitro fertilization (IVF)?

- o Yes
- o No

Have you or your partner used pre-implantation genetic diagnosis (PGD)?

- o Yes
- o No

Do you have adopted children?

- o Yes
- o No

If yes, did you adopt to eliminate the risk of a *CDH1* mutation in your children?

- o Yes
- o No

ATTITUDE TOWARD PRE-IMPLANTATION GENETIC DIAGNOSIS (PGD):

Do you want to have (more) biological children now or in the future?

- Yes
- No

Had you heard about pre-implantation genetic diagnosis prior to participating in this survey?

- Yes
- No

If yes, from whom or where did you hear about pre-implantation genetic diagnosis (PGD)?

- Physician
- Genetic counselor
- Family member
- Friend
- Internet or newspaper
- Other:_____________

Do you believe it is an acceptable practice for healthcare providers to inform individuals who have *CDH1* gene mutations about the availability of PGD?

- Yes
- No

If no, please describe the reason for your opposition:____

Have you previously considered using pre-implantation genetic diagnosis (PGD)?

- Yes
- No

If yes, did you meet with a reproductive endocrinologist to discuss PGD?

- Yes
- No

Please indicate your level of interest in learning more about PGD:

- Not interested
- Uncertain
- Somewhat interested
- Very interested

In general, do you believe that pre-implantation genetic diagnosis (PGD) is acceptable for conditions that occur during childhood?

- Yes
- No

Do you believe that pre-implantation genetic diagnosis (PGD) is acceptable for families with *CDH1* gene mutations?

- Yes
- No

If you want to have (more) *biological* children in the future, please rate the likelihood of you using PGD for the *CDH1* gene mutation in the future:

- o Definitely not
- o Probably not
- o Do not know
- o Probably
- o Definitely

If you answered 'definitely not' or 'probably not,' please explain the reason for your opposition:

____

If you have already completed your family, would you have considered using PGD for the *CDH1* gene mutation if this technology had been available when you were having children?

- o Definitely not
- o Probably not
- o Do not know
- o Probably
- o Definitely

If you answered 'definitely not' or 'probably not,' please explain the reason for your opposition:

____

Have any of your family members (other than your partner) previously used reproductive technologies?

- o Yes
- o No

If yes, which technology was used? (Circle all that apply)

- o Donor Gametes
- o In-vitro Fertilization (IVF)
- o Pre-implantation Genetic Diagnosis (PGD)

After completing this survey, will you discuss the availability of the PGD with a family member who has a risk to have a child with the *CDH1* gene mutation?

- o Definitely not
- o Probably not
- o Do not know
- o Probably
- o Definitely
- o Not applicable—nobody appropriate in my family

If you have any, what is your main concern or worry about PGD?____

____

If you believe there are any, what is the main benefit of PGD? ____

____

IMPACT OF HEREDITARY DIFFUSE GASTRIC CANCER (HDGC) SYNDROME ON LIFE:

How often do you worry about your chances of developing gastric and/or breast cancer (again)?

- o Not at all/Rarely
- o Sometimes
- o Often
- o Almost all the time

On a scale of 1 to 10 (1=not severe, 10=most severe), please rate how severely HDGC syndrome has affected your overall health and well-being:

0 1 2 3 4 5 6 7 8 9 10

Do you experience guilt related to HDGC syndrome affecting your family members?

- o Yes
- o No

How satisfied are you with your quality of life?

- o Uncertain
- o Not at all
- o A little bit
- o Somewhat
- o Quite a bit
- o Very much

Has having HDGC syndrome caused you to alter important life decisions?

- o Uncertain
- o Not at all
- o A little bit
- o Somewhat
- o Quite a bit
- o Very much

If you would like to tell us more about how HDGC syndrome has caused you to alter important life decisions, please do so here: ____

____

Has having HDGC syndrome affected your current or future reproductive decisions?

- o Uncertain
- o Not at all
- o A little bit
- o Somewhat
- o Quite a bit
- o Very much

If you would like to tell us more about how HDGC syndrome has affected your current or future reproductive decisions, please do so here: ____

____
